# An eDiary App Approach for Collecting Physiological Sensor Data from Wearables together with Subjective Observations and Emotions

**DOI:** 10.3390/s22166120

**Published:** 2022-08-16

**Authors:** Andreas Petutschnig, Steffen Reichel, Kristýna Měchurová, Bernd Resch

**Affiliations:** 1 Department of Geoinformatics, University of Salzburg, 5020 Salzburg, Austria; 2 Spatial Services GmbH, 5020 Salzburg, Austria; 3 Center for Geographic Analysis, Harvard University, Cambridge, MA 02138, USA

**Keywords:** urban analytics, mobile sensing, traffic, Participartory planning

## Abstract

Field measurement campaigns with traffic participants using wearable sensors and questionnaires can be challenging to carry out because of inconsistent interfaces across manufacturers for accessing sensor data and campaign-specific questionnaire contents bear large potential for errors. We present an app able to consolidate data from multiple technical sensors and questionnaires. The functionality includes providing feedback for correct sensor platform mounting, accessing and storing all sensor and questionnaire data in a uniform data structure. To do this, the app implements a sensor data bus class that unifies data from technical sensors and questionnaires. The app can also be extended to accommodate other sensor platforms provided they have a suitable API. We also describe a database structure holding the data from multiple campaigns and test subjects in a privacy preserving fashion. Finally, we identify some potential issues and hints that practitioners may encounter when conducting a measurement campaign.

## 1. Introduction

Contemporary urban planning approaches often seek to assess and improve traffic solutions that promote modal equity of traffic participants [1,2]. In many cases, however, implementing such solutions is a challenge. Decades of car-centric planning have often exhausted the limited amount of space that cities can allocate for infrastructure, leaving little room for active transportation. Reclaiming such contested spaces is notoriously hard because reshaping them not only leads to political friction, but is also expensive [3]. Especially for urban planning in an active transportation context, the subjective impression of traffic participants is an important decision criterion. One frequently used way of obtaining such insights is the use of quantitative methods based on questionnaires. These are, however, resource intensive to obtain and are therefore not well suited as a sole data source for modeling entire cities in appropriate scale. Thus, new approaches of assessing citizens’ perceptions and feelings about their environment are needed to create a more scalable and easy-to-use way of generating new insights into urban processes [4].

In this paper, we present a technical solution addressing the problems emerging from these circumstances. Specifically, we detail a mobile application for data collection in two use cases in an urban environment. The first one assesses whether an intervention has had the desired impact on traffic participants based on objective measures. For the second one, the challenge is not the assessment of a concrete measure, but rather the identification of potential candidate locations for planned interventions. Such a comprehensive overview, however, requires a more wide-ranging assessment of the traffic situation. What these use cases have in common is that ultimately planners seek to add the most value to an urban system with their limited resources. They therefore benefit from ways to objectively determine the merits of planned or already implemented interventions. An implementation of the concepts outlined in this paper can be found in the Supplementary Materials.

### 1.1. Concept and Terminology

The eDiary app presented in this paper serves two main purposes: (1) to collect and store physiological measurements provided by a variety of technical sensors, which are, for instance, connected to a smart device using Bluetooth; (2) to collect and store participants’ manual, individual inputs by means of a microsurvey.

#### 1.1.1. People-as-Sensors Concept

In this regard, the People-as-Sensors (PAS) [5,6] concept seeks to combine multiple data sources such as physiological sensor measurements and other relevant data to gain insights into a person’s perception of their surroundings. In an urban environment, wearable sensors can objectively measure the physiological response of what people experience in their subjective roles as traffic participants. By continuously monitoring a test subject’s physiological responses and location, we can therefore derive information about states of stress and relaxation that serve as indicators of traffic-related events and conditions. Additional, context-specific input from the person can help to further substantiate findings based on the technical sensor data. This combination of unprompted continuous data streams and prompted input from test subjects allows us to cover large areas of interest with highly granular and reliable information about traffic experiences.

#### 1.1.2. eDiary App Functionality

In this case, we utilize the concept to collect sensor data in a quasi-continuous and location-independent setting, i.e., “on the go”. Inputs may either be entered spontaneously by the test person whenever they encounter a situation that is relevant to the study, or they are prompted by the system in regular or irregular intervals or according to pre-defined rules (time-based, location-based, based on changes in physiological measurement data, etc.). The electronic diary (eDiary) app presented in this manuscript implements the data collection and processing functions necessary for the PAS concept by enabling geolocated in situ measurements from traffic participants. The typical modes of transport in our field studies that eDiary is designed for are walking or cycling, but eDiary can also be used in non-mobile settings. For example, for tests on a stationary bike in case measurements within a controlled environment are of interest. This can be the case for studies with the aim of benchmarking different sensors [7] or identifying the signal pattern of a moment of stress by exposing participants to a stressor or other stimuli and measuring the subsequent changes in their physiological signals [8,9].

A persons’ physiological signals alone, however, are insufficient for assessing their perceived safety. Active traffic participants are subject to a range of environmental influences such as the ambient temperature, rain or wind which, in combination with their physical constitution, affect the level of exertion and are therefore reflected in different physiological signals. Therefore, the signatures of indicators of stress moments in the data can be similar to other, irrelevant effects. For example, a sudden rise in a cyclist’s heart rate can be caused by experiencing stress, but also by a change in the street’s slope. Such effects can be discovered by integrating additional data in the form of questionnaires that the eDiary app provides. This way, stress moment candidates identified in the physiological data can be verified or rejected by the participants. Optionally, video footage from a helmet camera can help interpreting the sensor data.

#### 1.1.3. Terminology

A major part of the eDiary app functionality is to consolidate sensor data. When describing different aspects of the sensor measurement process, we adhere to the nomenclature from the Open Geospatial Consortium (OGC) Sensor Web Enablement (SWE) standards. The following definitions are used throughout the paper and are based on the OGC Sensor Model Language standard terms and definitions [10] reproduced here.

- **Sensor Platform (SP)** “An entity to which can be attached sensors or other platforms. A platform has an associated local coordinate reference frame that can be referenced relative to an external coordinate reference frame and to which the reference frames of attached sensors and platforms can be referenced.”
- **Phenomenon** “A physical state that can be observed and its properties measured.”
- **Sensor** “An entity capable of observing a phenomenon and returning an observed value. Type of observation procedure that provides the estimated value of an observed property at its output.”
- **Measurement** “An observation whose result is a measure.”
- **Measure** “Value described using a numeric amount with a scale or using a scalar reference system.”

## 2. Related Work

The eDiary apps are used for collecting a person’s feedback to a phenomenon under investigation, oftentimes used to monitor patients or study participants over extended periods of time in medical settings [11,12,13]. One motivation behind using this form of data acquisition is that it can be specifically tailored to use in a given use case by collecting data from different technical sensors. An app can also prompt participants to perform tasks and to report feedback [14]. Once implemented, studies using eDiary apps can be cost-effective and scalable [15] if the participants can use the app on their own. There is therefore little need for input from researchers during the data collection phase. This, however, requires the app to be accessible to the users. Specifically, the app has to be easy to navigate, utilize colors appropriately, provide adequately sized menu buttons and easy-to-interpret messages [16]. Finally, a strongly related effort, a People-as-Sensors app, which this paper is based on, has been presented previously [17], which builds the conceptual basis for the eDiary app presented in this paper.

## 3. eDiary App Requirements

### 3.1. Data Acquisition

As the eDiary app will consolidate physiological sensor measurements and survey inputs as part of the PAS concept, we identify three principal requirements for the app functionality.

1. For sensor measurements, the app needs to connect to multiple SPs and process their data.
2. For surveys, the app needs to provide a questionnaire view that can be adjusted to a given use case.
3. While the measurements are running, the app needs to provide feedback to the user about the sensor connectivity status.

1: The app must be able to handle multiple SPs as long as they provide access to the measured raw data via an application programming interface (API) from the vendor that provides functions for accessing the data programmatically. Depending on the type of sensor, the data can have different data types. The app must therefore support primitive data types and character strings for the sensor data. The app’s code base must also be designed in a way that allows the integration of new SPs with minimal changes to the source code.

2: The question and answer possibilities in the user microquestionnaire are often strongly dependent on the particular requirements of a study or use case. When conducting a new study, researchers need to be able to easily generate and adapt questions together with according response options that are presented to the user. For instance, studies in different cities or on different mobility modes (e.g., walking vs. cycling) will have different characteristics and requirements. Therefore, support for a simple, text-based configuration file that encodes interaction options and can be adapted without recompiling the app is required. Similarly, the questionnaire (questions, question types, answer possibilities) can be easily adapted by non-technical experts.

3: Test persons wearing an SP often need to adjust the fit of wristbands or chest straps to ensure valid sensor readings. Visual information in the form of the real-time sensor readings are an easily interpretable feedback mechanism for users to help them mount all sensors properly.

### 3.2. Data Structure

The app needs to store the collected data in a way that accommodates the following aspects:

1. Integration of physiological measurements from multiple SPs in a unified format.
2. Storage of subjective inputs and physiological measurements.
3. Integration of different units of measurements and sampling frequencies.

1: Data coming from different SPs and sensors must be stored in a consistent format. Each measured datum stored by the app must be linked to a timestamp and information about the SP and sensor. The data structure must be consistent for all measurements, regardless of which SP and sensors are used.

2: Like the measurement data, inputs from the users must be stored consistently as well. This means that all user input data must be compatible with the data model implemented in a central database.

3: Different sensors vary in their sampling frequencies. Data with ≤1 Hz frequency are stored as individual rows, higher frequency data can also be concatenated to strings or aggregated within the eDiary app and then stored in a data type compatible with the aggregate.

## 4. Methods: Design and Implementation of the eDiary App

This section transparently lays out the app’s architecture and core components, together with a justification of design and implementation choices. This includes the general app design with a focus on the mobile back-end system, the data acquisition process, the data model for storing the data, and the app’s front end, complemented by some privacy considerations and data-handling procedures.

### 4.1. Data Acquisition Process

The phenomenon under investigation—a person’s physical responses to the environment—are measured using sensors. We used two SPs from commercial manufacturers in our experimental settings. The first one is the Empatica E4, a wristband that features sensors to measure, among others, blood volume pulse (BVP), galvanic skin response (GSR), inter-beat interval (IBI) and skin temperature (ST) [18]. The second one is the Zephyr BioHarness 3, which consists of a chest strap with an electronics module with two electrodes mounted on it. It delivers two-point electrocardiographic measurements and positional parameters [19]. The requirements for the smart phones running the app are the operating system Android version 8.0.0 (API version 26), Bluetooth connectivity and a global navigation satellite system (GNSS) sensor. We conducted our studies using Samsung Galaxy A3 phones.

Table 1 shows a list of all available SPs and sensors in this configuration. The sensor availability partly overlaps, but there are also sensors that are inactive, meaning that they are not accessible via the API and are therefore not usable for the eDiary app. The mobile phone is used for obtaining time and location information and for temporary data storage. During the data acquisition process, the app establishes a connection to the SP, stores measured data and provides visual feedback to the user on the connection status of all linked SPs.

### 4.2. Data Storage

**Figure 1:**
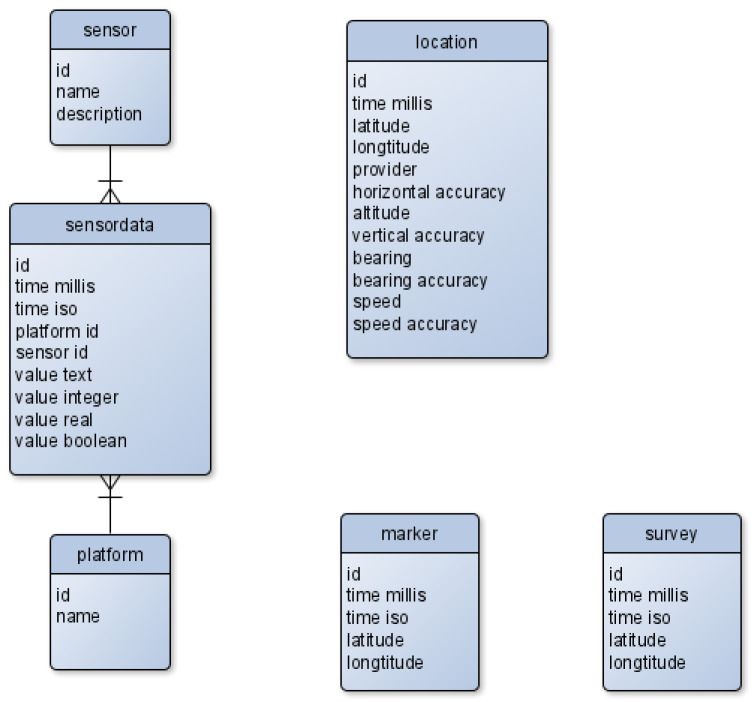
Entity relationship diagram SQLite database.

**Figure 2:**
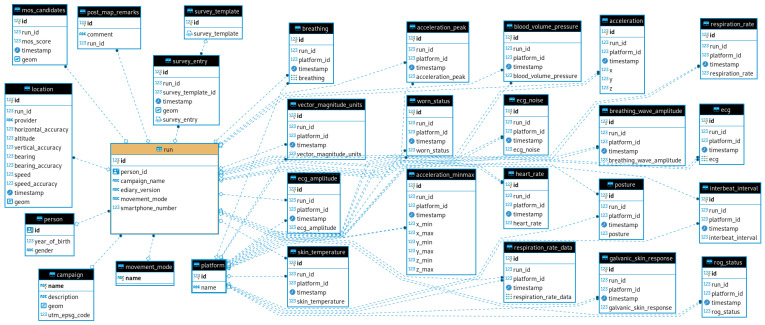
PostgreSQL database schema designed to hold data from multiple measurement campaigns, users and sensors.

During data acquisition, all data are stored locally in a SQLite database. After the acquisition is completed, the data is imported into a PostgreSQL database that holds measurements from all test runs, sensor information and other metadata. From there, it is possible to manage data access, create views and aggregates of the data and feed it into scripts for further analysis. The SQLite schema in Figure 1 captures the position, timestamp and sensor measurements, along with the type of sensor and SP. In addition to the measurement data, it also has a table “marker” that contains unspecified points that can be generated by tapping a button in the user interface (UI). The purpose of these markers is to provide an uncomplicated way for the user to mark and re-identify anything potentially relevant during the measurement, which is common practice in numerous disciplines including psychology, medical science other fields. The more elaborate schema on the server side PostgreSQL database shown in Figure 2 allow storing data from multiple measurement campaigns. In addition to the data from the SQLite files, it contains information about the participants and the measurement campaigns. The data structure uses the measurement and runs as the central point connecting persons to locations, measurements and survey inputs. It therefore enables access to individual measurement runs or sensors and also to data over multiple runs from specific persons or other arbitrary criteria, thereby covering a wide range of use cases.

### 4.3. Mobile Back End

#### 4.3.1. Sensor Platform Connectivity

The app was designed to work with SPs that use Bluetooth for data transmission and expose functions for accessing data via a Java API. For this functionality to work, the eDiary app has to implement functions that facilitate sensor connectivity. The initial pairing between an SP and a smartphone is performed via the smartphone’s built-in Bluetooth functionality. After this step, a function provided by the SP API can discover the paired SP. If an SP is found, the API methods for accessing data become available, and measurement data can be accessed accordingly. In a controlled setting, this is the only step necessary to conduct measurements. However, in field tests, smartphones and sensors may be stored in different pockets or bags, and they move around during physical activities. This can lead to connection loss between the devices. To mitigate this, the API or eDiary app has to provide functionality to monitor the connection status of the SP and, if necessary, make reconnection attempts. The eDiary app also provides visual feedback to the user to inform about the connection status so they can intervene if the app fails to reestablish a connection automatically. At the end of the measurement session, the pairing can be broken up again via an API call.

#### 4.3.2. Sensor Data Bus

The functions provided by the SP APIs for data access are not consistent throughout manufacturers and products. Data access functions may expose data as primitives or composite data types, have different names for the same functionality or provide new data in different intervals. To facilitate the required data access functionality, the eDiary app needs to consolidate all incoming data. The SensorDataBus class in combination with SensorDataCollection and SensorDataEntry objects implements this functionality. The SensorDataBus therefore acts as a broker for measurements between the different SensorPlatform objects and components that register themselves with a SensorDataBusListener via the SensorDataBus. After having received sensor data, the eDiary app creates a SensorDataEntry object containing the data and an ID specifying the sensor. This step also includes all sensor-specific data parsing and error handling. One or multiple SensorDataEntry objects along with timestamps and an SP ID then go into one SensorDataCollection object, which is then passed to the SensorDataBus. SensorDataBus exposes a listener function for incoming data to which other functions in the code can subscribe. Each of the registered listeners is informed about new data in the onDataAvailable() method of the SensorDataBusListener interface. The notification of the listeners is executed in an individual thread for each registered listener. This way, long-lasting tasks in one listener will not block the notification of the other registered listeners. Within a listener, notifications are queued until the handling of the previous notification is finished.

**Figure 3:**
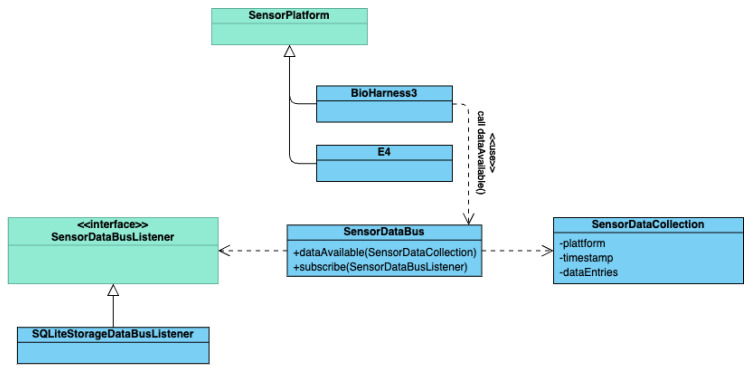
Schematic overview of eDiary data acquisition process.

The advantage of this method is that a function that requires data has access to all incoming data in the same format. It also ensures that all functions receive exactly the same data packages from all SPs, which would not necessarily be the case if they would receive data directly from the sensor APIs. In addition to good usability and data integrity, the implementation also promotes maintainability of the app because any sensor specific code can be assumed to be handled before being passed to the SensorDataBus in the program flow and not scattered throughout the source code. Figure 3 shows the schematic overview of the data flow from observation to stored data.

#### 4.3.3. Sensor Data Export Functions

The data collected by the eDiary app are written into a database. At the beginning of a measurement session, the app creates an SQLite database with a table that can store a measurement’s timestamp, the SP, sensor and value. The Recording class subscribes to the SensorDataBus listener and receives all data from incoming SensorDataCollection while running in the background. To avoid database overhead, Recording caches 100 SensorDataCollection objects before writing them to the database. If the measurement session is stopped, all remaining cached SensorDataCollection objects are written into the database.

#### 4.3.4. Storing Measurement Variables

The eDiary app can deal with multiple SPs. However, it is possible that not all SPs are used during a run. The data structure of the resulting database should always be the same, independent of which SPs and sensors are used during a run. This way, subsequent data analysis workflows can always assume the same structure for all input data. We accomplish this sensor agnostic storage schema by providing individual columns for data types as shown in Table 2. This schema can also deal with sensors that are activated after a measurement run has started, which may sometimes be necessary due to connection issues of individual platforms.

#### 4.3.5. Handling New Sensor Platforms

When adding a new SP to the eDiary app, the platform manufacturer has to provide the libraries that facilitate the interaction between smartphone and platform. Based on the libraries’ data retrieval functions, we can implement a new platform-specific class that inherits the SensorPlatform class. This class, in turn, can implement functions for retrieving data from all sensors. During operation, it is irrelevant which SPs are connected to the phone when starting the app or in which order they are added. Additionally, the SensorManager class has to implement an additional intent for the respective platform, so the platform can be reached via the startIntentServices method in SensorManager. To handle the platform on the Android side, AndroidManifest.xml needs to be extended with an additional <service> element. Finally, the SensorPlatform object can pass data to other components of the app via the SensorDataBus.

#### 4.3.6. Handling Different Sampling Rates

The SensorDataBus can handle different sampling rates because every sensor measurement is stored independently of the other measurements. Measurements can either be passed on to data storage as they are or, if the SP sends a set of values of high frequency measurements, can optionally be aggregated beforehand. The app also records location data using the phone’s positioning functionality. The individual sensor measurements and locations can be joined in postprocessing based on their timestamps to establish the link between geographic location and physiological measurement. In experimental settings, the phone’s GNSS sampling frequency of 1 Hz was adequate to position the SP data.

### 4.4. App Front End

**Figure 4:**
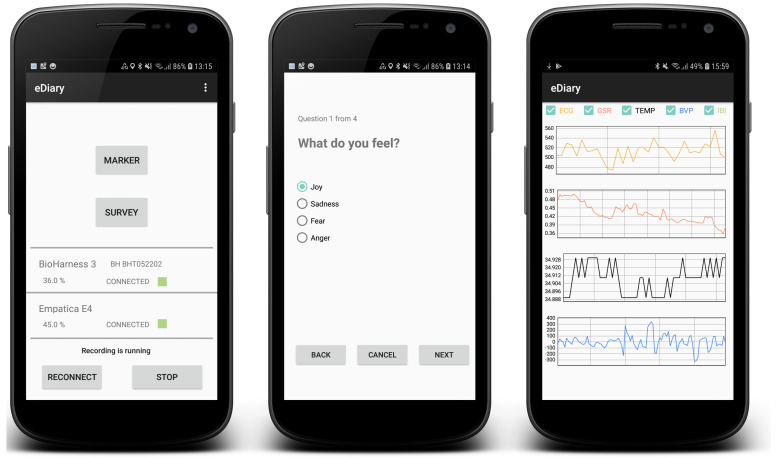
Overview of the eDiary UI.

Figure 4 shows different aspects of the the eDiary app UI that help the user check the measurements running and interact with questionnaires. All user interaction elements are easy to reach and labeled clearly to make the app easy to use for a wide range of users.

#### 4.4.1. Sensor Platform Connection Status/Reconnect

The base functionality of the eDiary app is to connect to the SP and store the data. To ensure these functions are carried out, there are only a few front-end functions required. A status monitor for the SP gives information about the connectivity status of the connected devices. In case of any unexpected errors regarding any other app functions, it provides error messages as pop-up messages.

#### 4.4.2. UI for Data Input

The button labelled “Marker” allows the user to store the current timestamp and location without any further interaction. This can be useful if the user wants to give further input regarding the location after finishing the measurement run. The “Survey” button leads the user to a menu in which they can select predefined answers to questions. The questions and possible answers can be adapted via an XML file that the eDiary app sources on startup.

#### 4.4.3. Data Visualization

Real-time sensor data graphs are not required for the measurement process itself but provide feedback to the app user when adjusting the measurement device. They also give users the opportunity to test if certain movements disrupt the measurement process and adjust accordingly. The functionality is therefore an important tool for data quality assurance at the first step of the data-handling workflow. The visual impression of the measured data in real time is also an easily interpretable way of presenting the direct link between physical or psychological activity and physiological data feedback. This makes the data visualization a useful tool for presentation and teaching purposes.

### 4.5. Sensor Platform Requirements

Conceptually, the functionality of the eDiary app is independent of the manufacturers and types of used SPs. To implement the app in a practically feasible way, however, the sensor platforms need to fulfill certain requirements apart from being accessible via Bluetooth. The SP must provide an API that enables the App to communicate with the SP and retrieve the collected data in real time. Alternatively—if no suitable API is available—the communication protocol and data formats of the SP must be documented so that the app itself can implement the API.

### 4.6. Linking Location and Measurement Data

To link the sensor measurements to a location, we use the timestamps collected along with the locations to the closest measurement. This step is performed in postprocessing inside an indexed database and not in the eDiary app to avoid computationally expensive processing on the smartphone. If there are no GNSS data available for a measurement run, as is the case in indoor settings, the sensor data are not linked to any location.

### 4.7. Privacy by Design Considerations

After participating in a measurement campaign, the participant might wish to have their data deleted. However, if for privacy reasons, the measurement data are not linked to any identifiable characteristics of a specific person, it is not possible to identify and delete the correct data to delete. One way of handling this task is pseudonymization. This can be achieved by linking the data to the digest of a cryptographic hash function based on their identifiable data. For example, for a fictional user named Jane Doe, born on 1 January 1970, we can concatenate the input data to the string JaneDoe1970-01-01. Passing this string to a MD5 hash function produces the digest 682f93217364bff061f89f85f2e4fd4d, which can be used as a secure identifier that allows reidentification of a known person.

## 5. Discussion and Limitations

This section summarises and discusses recent experiences from different field studies in which the eDiary app was used to collect data in real-world environments and then critically reviews a number of limitations of design decisions, technical and technological aspects, and the app’s usage in the field.

### 5.1. Experience from Recent Field Studies

**Figure 5:**
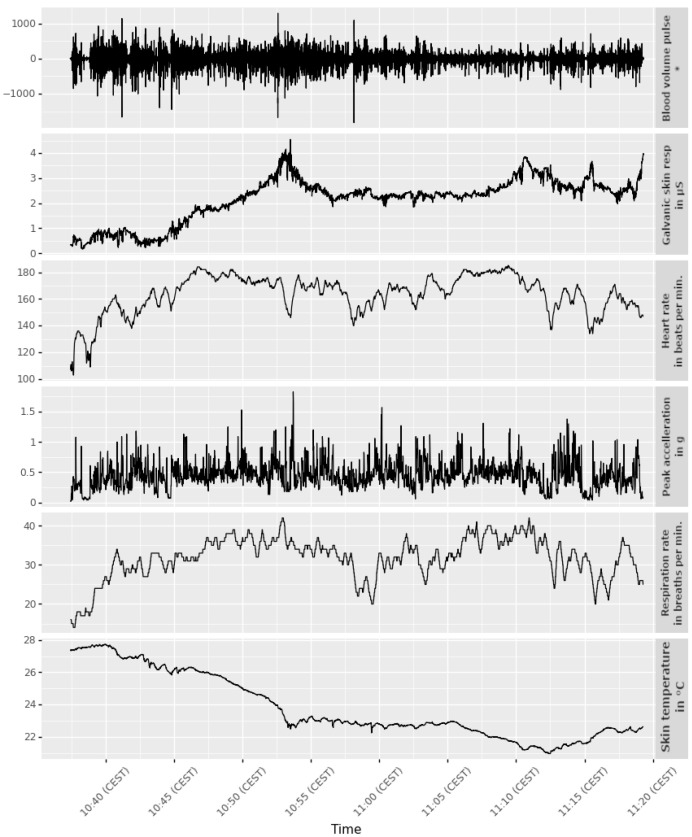
Example of measured physiological data. The asterisk in the subplot of the blood volume pulse indicates that this is a unitless measure.

**Figure 6:**
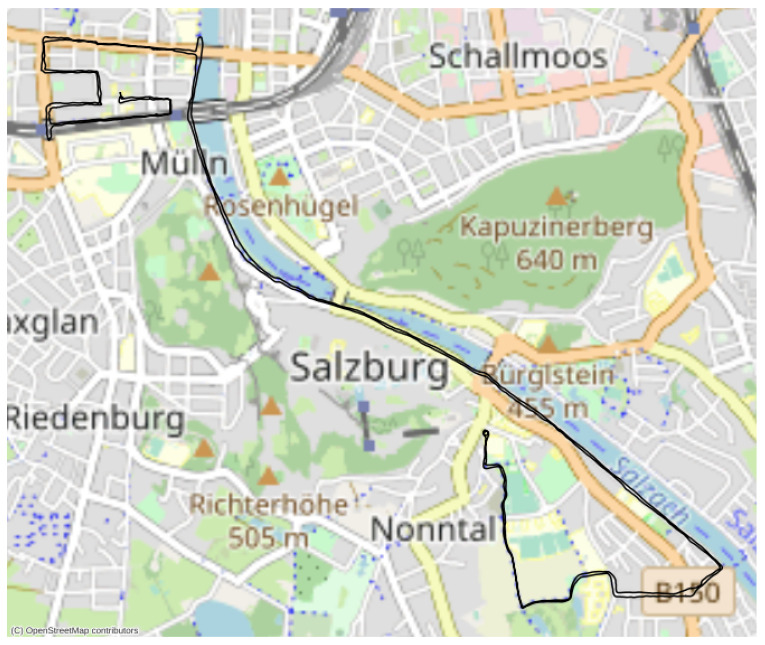
Location data from a test run plotted on a map.

The eDiary app has been used successfully in multiple studies and worked consistently in the field. In one project, the app was used to collect data from over 250 participants over the course of one year in Salzburg, Austria. Figure 5 and Figure 6 show examples of physiological and location data collected during these studies. The particular example was collected by a cyclist participating in a study that investigated perceived stress while cycling in the city of Salzburg, Austria. The person was equipped with the E4 and BioHarness, the measurement app was started, and the person cycled independently on a pre-defined route from the starting point in the south (Unipark Nonntal) to the end point in the north (close to S-Bahn Mülln-Altstadt). The route was chosen to contain different sections that are differently challenging for a cyclist—e.g., parts with low traffic, parts where there is a lot of bicycle traffic and parts with more car traffic.

It may happen that some of the collected data are erroneous, for example because a sensor slipped due to movement during the measurement. Such inconsistent errors are hard to detect. Therefore, to keep the complexity of the app low, there is no check for outlier values on recording. All sanity checks are performed in a later analysis stage via exploratory statistics. These include comparing the length of a run according to the recorded locations vs. recorded physiological data, checking if the sampling frequency is as expected, identifying unrealistically low or high outliers in the data and visually interpreting the measurements.

### 5.2. Recommendations for Data Storage

An alternative to storing measurement data first on a local SQLite database and then transferring it over to another database that contains data from all measurement runs is to transfer the data on the fly. Possible solutions for transferring sensor and location data are the OGC Sensor Observation Service [20] standard or the MQTT network protocol [21]. These and similar technologies, however, have the drawback of requiring a mobile network connection. In some of our study areas this is not feasible, which is why we resort to local storage with the additional data transfer step.

### 5.3. Discussion of Requirements

#### 5.3.1. Data Acquisition

For data acquisition, the required functionality of the app is the support of multiple SPs, the availability of questionnaires and the feedback to the user about sensor connectivity. Requirement 1 is fulfilled by implementing a generic SensorDataBus that is able to handle all SPs as long as they provide an API that exposes functions for accessing the raw data and are accessible via Bluetooth. Requirement 2 is fulfilled by providing questionnaires that are easy to customize to the user via the UI. For customizing questions and response options, the researchers have to manually edit an external xml file that is stored on the phone and has to adhere to a predefined structure. A possible future improvement would be the option of editing the questionnaire directly via the app. Requirement 3 is fulfilled by showing the current connectivity as text and color coded and the battery status as the percentage of all connected SPs on the main view of the UI. Additionally, recent measurements of selected sensors can be displayed as graphs. Currently, the sensors to be displayed are hard coded in the app. An improvement would be to allow the user to select which ones to display.

#### 5.3.2. Data Structure

We addressed requirements 1 and 2 for data storage by designing the data model in a way that allows us to store all collected data in a consistent structure regardless of which combinations of SPs and sensors are used in a measurement campaign or which questions and answers are available. For the unprompted sensor data, this approach covers all use cases that we encountered in a wide range of practical use cases. In the case of prompted user input in the form of questionnaires, our approach requires researchers to model their questions and response options as simple pairs of question and answer. Requirement 3 is solved by storing each collected datum with a timestamp with a one-second accuracy. In case of sensors that collect data with >1 Hz, an aggregation scheme has to be implemented in the source code of the eDiary app.

### 5.4. Practical Hints

#### 5.4.1. API Dependencies

Some SPs or their APIs have specific characteristics that may be unexpected and have to be addressed before they can be used. For example, one SP used in our settings requires the linked smartphone to provide Internet connectivity for authentication of the device. Only after authentication, the device can be used for measurements. This means that this SP cannot be used in areas with no mobile Internet reception or if the smartphone is not equipped with a SIM card. This particular shortcoming can only be addressed by utilizing an SP that also works without an active Internet connection.

#### 5.4.2. Logging

The eDiary app is a complex piece of software that combines multiple hardware components via their individual APIs. Much of the functionality also relies on an active Bluetooth connection between the individual actors. These factors make it challenging to identify and reproduce erroneous behavior of the app. It is therefore useful to write potentially useful logging information to a file during regular measurement campaigns. This way previously unknown bugs that may only be triggered by certain user behavior or unexpected environmental influences can be detected, documented and fixed.

#### 5.4.3. Timestamps from Multiple Devices

Most SPs provide not only sensor readings via their API, but also the corresponding timestamp. This is useful because we not only store the sensor data, but also need the temporal information for analysis. However, when multiple measurement devices are in use, users have to make sure that all devices’ internal clocks are synchronized prior to the measurement session to ensure that there is no systematic lag between the different SPs. Doing so is time-consuming and error-prone because it involves numerous manual steps on different UIs. To eliminate this problem, the eDiary app strictly uses timestamps generated by the smartphone upon receiving sensor data from its internal clock. This way there is only one source of systematic error for timestamps, and if an error occurs, it can easily be corrected for all data of a measurement session. An error is also very unlikely to happen provided the smartphone is able to synchronize its internal clock via the cellphone network. One drawback of this method is that all timestamps mark the time when the app received a datum and not when the datum was actually captured. Although we see the error introduced by this lag as largely negligible, a possible solution to this issue would be to set the internal clocks of the SPs on connection to the time of the phone via an API call and then use the timestamps that the SPs send along with the measurements.

#### 5.4.4. Time Zones

When conducting measurement campaigns in different regions and times of the year, the time zones of the measurements are of importance when joining data to avoid systematic time errors in the result. The Android platform provides methods to handle time zones in the java.util.Calender class. In our data structure, all measurements are stored in the UTC time zones, so comparing data sets from different time zones is possible, but local times can also be easily reconstructed.

## 6. Conclusion and Outlook

### 6.1. Linking PAS and Other Data

We were able to fulfill the requirements of our study designs using the detailed app. The most critical requirements for this assessment were accessing and storing the collected data and giving the user easily interpretable feedback about whether the sensor platform is switched on and connected to the app. Also of great importance was the functionality facilitating the customizable questionnaires. Without these requirements, the app would not be fit for use in our studies.

Less critical features that are not strictly necessary for the utility of the app in field studies but are nevertheless useful by making the data collection more robust and the later data analysis workflow more efficient are the real-time visualization of data streams and storing the data in a unified format, independent of the sensor platform and data type of the measurement. By adding these features to the app, we lower the chance of erroneous data caused by inappropriately mounted sensor platforms or by manual post data postprocessing.

After vigorously testing the app in the field, we can conclude that the app not only fulfills our requirements in theory, but also proved to be useful and applicable in real-world conditions. However, there is still room for improvement and extensions when considering different study designs. An important next step would be linking the data collected via the PAS app with other data. Physiological measurements and questionnaires are not the only ways of gaining insight into the human perception of urban settings. Latent information, for example derived from the contents of unstructured data sources such as georeferenced social media data, newspaper articles or official data sources such as accident statistics, can be used to enrich physiological measurements. However, differences in scale, purpose of the data and data structure make a useful union of such diverse data sources challenging. Even though such challenges are not at the core of the eDiary app itself, they can potentially influence future design decisions on data modeling and selection of the SP.

### 6.2. Additional Data Sources

On most measurement campaigns, we also acquire video data by using an action camera (e.g., GoPro). At the moment, those videos have to be analysed manually to extract the source of detected MOS (such as a car overtaking with very little distance). For automatic frame extraction, the videos and data streams have to be aligned manually at the moment. A good method for semi-automatic alignment of both has to be developed. In the future it might be desirable to develop a fully automatic method of synchronizing video and sensor measurements.

In a recent study [22], the data collected by the eDiary app (physiological sensor data and eDiary entries together with GNSS positions and timestamps) were integrated with each other to assess the bikeability and walkability of different cities. The produced information (hot spots and cold spots, i.e., areas of stress and areas of relaxation) was then validated with first-person videos collected through chest-mounted cameras and georeferenced post hoc questionnaires.

### 6.3. Beyond Field Studies

The majority of field studies conducted with the PAS app took place in Salzburg, Austria, which is a fairly small city with appropriate bicycle infrastructure. When applying the above study designs to other less bike friendly environments, the appropriateness of conducting a field study has to be evaluated on a case-by-case basis, ideally with the help of local traffic experts. If a field study is not possible, one alternative is to perform the study in a virtual environment which has to be recorded only once. In one study [23], eDiary entries and physiological sensor data were analysed to identify hot spots and cold spots, which were then compared for physical and virtual environments. The study that took place in the physical environment (i.e., in a real-world urban setting) was carried out following the study design of a preceding study [4]. For the virtual study, a real-world track was recorded using a 3D camera, and the resulting 3D video was then played to test persons in virtual reality glasses in a lab setting. The goal was then to compare moments of stress and relaxation between the real and virtual environments.

## Figures and Tables

**Table 1:** Complete list of platforms and measurements available used in this study.

Platform	Measurement	Active
Zephyr BioHarness 3	Acceleration data	No
Zephyr BioHarness 3	Breathing	Yes
Zephyr BioHarness 3	Breathing wave amplitude	Yes
Zephyr BioHarness 3	Electrocardiogram (ECG)	Yes
Zephyr BioHarness 3	ECG amplitude	Yes
Zephyr BioHarness 3	ECG Noise	Yes
Zephyr BioHarness 3	Galvanic skin response (GSR)	No
Zephyr BioHarness 3	Heart rate	Yes
Zephyr BioHarness 3	Peak acceleration	Yes
Zephyr BioHarness 3	Posture	Yes
Zephyr BioHarness 3	Respiration rate	Yes
Zephyr BioHarness 3	ROG status (Fitness index—Red, Orange, Green)	Yes
Zephyr BioHarness 3	RR interval in QRS complex	Yes
Zephyr BioHarness 3	Skin temperature	No
Zephyr BioHarness 3	Vector magnitude units—activity measure (VMU)	Yes
Zephyr BioHarness 3	Worn status	Yes
Zephyr BioHarness 3	XYZ acceleration min and max	Yes
Empatica E4	Acceleration data	Yes
Empatica E4	Blood volume pulse (BVP)	Yes
Empatica E4	Galvanic skin response (GSR)	Yes
Empatica E4	Inter beat interval (IBI)	Yes
Empatica E4	Skin temperature	Yes
Smartphone	Location	Yes

**Table 2:** Schema of the sensor agnostic data storage.

Column Name	Description
ID	Unique Identifier
timestamp	Timestamp in UTC
sensor_platform	Primary key of the sensor platform
sensor	Primary key of the sensor
value_text	Data of type text
value_integer	Data of type integer
value_real	Data of type real
value_boolean	Data of type boolean

## Supplementary Materials

The following are available at: https://www.mdpi.com/article/10.3390/s22166120/s1, PAS App source code.
