# Supplementary material for: An eDiary App Approach for Collecting Physiological Sensor Data from Wearables together with Subjective Observations and Emotions
Source: Sensors (Basel). 2022 Aug 16;22(16):6120. doi: 10.3390/s22166120 (PMC9414387; doi:10.3390/s22166120)
Supplement: Supplementary file 1 [file sensors-22-06120-s001.zip › supplementary_materials/v0.4.0_documentation.pdf]

Contents:

1 **User Guide** **2**
- Installation . . . 2
- Configuration File . . . 2
- Usage . . . 3
- Reconnecting the Sensor . . . 4
- Visualization . . . 4
- Marker Button . . . 4
- Output File Structure . . . 4

2 **Developer Guide** **5**
- Code Conventions . . . 5
- Checklist for new Version . . . 5
- Introduction . . . 5
- On starting the App . . . 6
- Sensors . . . 6
- SensorDataBus . . . 7
- Data Storage . . . 7
- Running Application in Emulator . . . 7
- Documentation . . . 8
- Loading Data from Smartphone to Computer . . . 8
- Remove application folder on device . . . 9
- Backup eDiary data directly on phone . . . 9
- Naming of the Phones . . . 9
- Request balance of phone . . . 9
- Switch Off Pin for SIM-card . . . 9
- BioHarness . . . 9

3 **Usage in Field** **9**
- Preparation . . . 10
- Data Organisation . . . 10
- GoPro . . . 10
- BioHarness . . . 11
- Empatica E4 . . . 11
- Phone . . . 11
- Storing the data later on . . . 11
- Miscellaneous . . . 12

4 **Changelog** **12**
- v.0.4.0 - 2020-01-21 . . . 12
- v0.3.0-alpha - 2019-04-10 . . . 13
- v0.2.0 - 2018-10-05 . . . 13
- v0.1.0 . . . 13

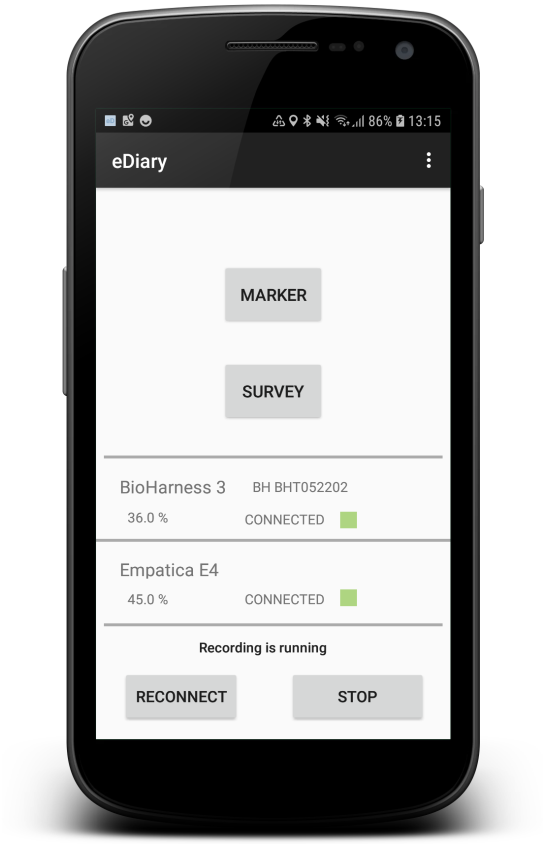

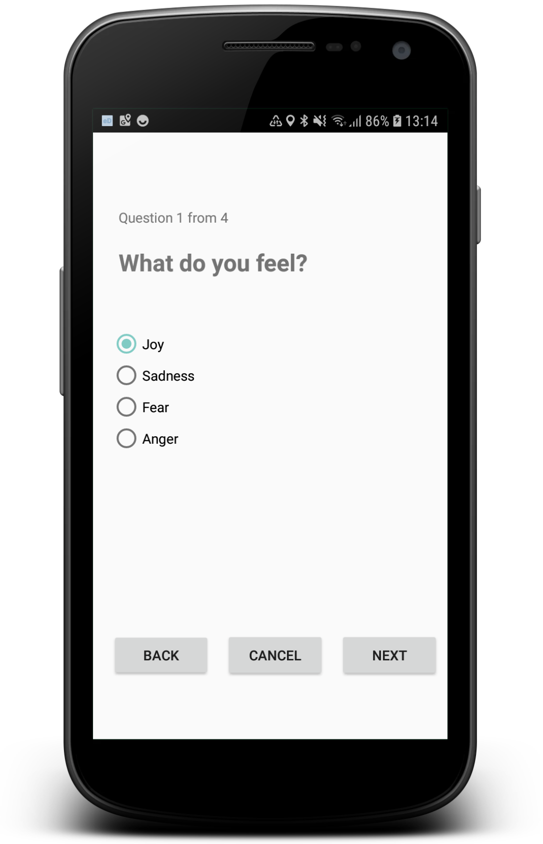

The PDF version of this documentation can be found here .

1 User Guide

This app only works with Android 8 - Oreo (API 26 / API 27)

Installation

To install the app transfer `eDiary.apk` to the smartphone and use a file manager of your choice to install it.

For the installation to work, it may be necessary to allow the installation of third party apps in the phone's developer options, which can be found in the settings menu.

Configuration File

Note: Modifying `config.xml` may lead to unexpected behavior. It is always possible automatically to create a default version of this file by manually deleting `config.xml` from the phone and restarting the app.

The file `config.xml` contains configuration parameters that are needed for the app to run. It has to be placed in the folder `eDiary` and can be adapted according to specific usage requirements.

The file features the following XML elements:

<UserInterface>

Allowed values for each item of `<UserInterface>` are `true` or `false`.

- `<SurveyIsActivated>`: If survey shall be activated or not.
- `<RealTimeVisualizationIsActivated>`: If the visualization of the real time data shall be activated or not. Default is *false*.
- `<MarkerButtonIsActivated>`: If a marker button shall be displayed. A marker stores the time and the location.

<Survey>

Definition of survey questions. There are three categories for `<SurveyItem>`s. Each of them needs a `<Question>`:

- `<RadioButtonGroup>` needs a set of `<Answers>`
- `<Slider>` needs a `<Maximum>` value, the minimal value is always `0`
- `<TextInput>` does not need any specification (except of the `<Question>`), the text length is not limited

<Sensors>

Definition of the sensor platforms for data collection.

The Empatica E4 sensors require authentication with an API key. To be able to use it to collect measurements with the eDiary app, the API key, which can be obtained on the manufacturer's website using the device's serial number, has to be entered in the `<Sensor>` element under `<API_key>`.

Usage

When first starting the app, it will once prompt the user to allow access to the phone's data storage and location. These permissions must be granted in order for the app to work.

Make sure the E4 device is properly registered on the E4 developer website. Otherwise it will not work.

Note: Internet access is necessary in order to connect to Empatica E4.

After the app has been started, make sure that the Empatica E4 sensors is connected. Turn on the sensor by clicking 2 second on its button, please pay attention to the LEDs:

- **Green**: ready
- **Light blue**: Bluetooth connection is available

- **Dark blue**: sending data via Bluetooth
- **Yellow**: battery is low or charging
- **Red**: recording has started

After 20 seconds, the small LED on the sensor turns off (for power safety reason). Check the sensor's connection to the smartphone (displayed on the main screen). Finally, please mount the sensor on your wrist, pull the wristband tight.

The recording starts when `Start` is clicked or a survey is submitted. The user can anytime submit a survey about the current emotion, context and intensity. The recording can be stopped by hitting the `Stop` button. All collected data can be found in the phone's folder in `eDiary`.

Reconnecting the Sensor

If the sensor connection breaks, please re-connect the sensor as follows. Restart the app or push the `Reconnect` button, then turn on the Empatica E4 device again. If the sensor is connected to the phone and you close the app, it will shut down the sensor as well.

Visualization

The real time visualization of the incoming sensor values can be accessed in the menu on the top right (if it is activated in the `config` file). **Warning**: This feature sometimes crashes. It should therefore be seen as **experimental**. It is deactivated by default, but can be activated by editing the configuration file.

Marker Button

The marker button registers the current time and the current location. The values will be stored in the table *marker*. Clicking on the marker button also starts the recording.

Output File Structure

The app writes all data in to a **SQLite Database:** It consists of the following tables:

- **platform**: Contains names and ids of all supported sensor platforms.
- **sensor**: Contains names, descriptions and ids of all sensors.
- **sensordata**: Contains timestamps, ids and values of all measurements stored on the database.
- **survey**: Contains timestamps, location and results of all survey entries
- **location**: the locations measured by the phone. With additional metadata like vertical accurracy, bearing, speed, . . .
- **marker**: the marker entries, if the function is activated

2 Developer Guide

Code Conventions

In order to keep the project clean and consistent some rules need to be respected:

- write proper **commit messages** and do only commit one logical change at once
- use the default **autoformat** tool from Android Studio in order to keep the code formatting consistent
- for versioning we use Semantic Versioning 2.0.0, hence tag the releases with `MAJOR.MINOR.PATCH`
- Additionally add the changes to the `CHANGELOG.md` file which is structured using Keep a Changelog.
- reference external libraries with Gradle and attribute them in the `AboutActivity.java`, also attribute snippets from StackOverFlow (see this blogpost)
- document all changes in the source code and check properly if you did not break anything (there are no unit tests yet). Also update both the user and the developer documentation and the `README.md`

Checklist for new Version

1. Make sure everything works well and no existing features have been broken.
2. Document the changes, remove unused documentation
3. Tag your commit via: `git tag v0.7.0; git push origin --tags;`
4. Also add the version number to the documentation.
5. Add the version number to the manifest of the app.
6. Write a CHANGELOG
7. Make a GitHub Release, attach the APK, the PDF of the documentation and a sample SQLite file with real data (necessary for testing)
8. Update screenshots for docs and README

Introduction

This document is supposed to help understand the way this app is designed and how the implementation of processes for data collection and storage work.

Note: All Java classes mentioned here are located in the package `at.ac.sbg.zgis`.

On starting the App

The entry point of the app is located in the class `userinterface.EDiary.java`. It starts the user interface (UI). Thereafter, the device starts searching for a GPS or network location.

Sensors

Starting Sensor Measurements

Each sensor class extends `sensors.SensorPlatform.java` and consequently also `IntentService`, which allows it to run in a separate thread. The `startIntentServices()` method initializes the `SensorPlatform` objects in the `SensorPlugin` class, which is in turn called from the method handling the *connect* action (`handleConnect()`).

Handling Sensor Measurements

`SensorDataCollection` objects contain all new sensor data, which comprise one or multiple `SensorDataEntry` objects. `SensorDataCollection` contains information about the sensor platform with which the data was collected and a timestamp. `SensorDataEntry` contains the specific type of sensor along with the measurement result.

The sensor handler classes pass all `SensorDataCollection` objects to the `SensorDataBus`. This class acts as a broker that hands data to other classes which can subscribe to it using the `SensorDataBusListener` interface. These classes can then perform tasks, like storing or visualizing the data.

Adding new Sensors

When adding a new sensor to the app, i.e., when integrating a new data protocol for sensor measurements, the manufacturer's SDK has to be included in the app's libraries. Then a new sensor class in the `sensors` package has to be created. To work properly in the app, it has to be a subclass of `sensors.SensorPlatform`. In this class, the collected data has to be passed to `SensorDataBus`. Also, new entries to the enums `SensorPlatform` and `Sensor` in `configuration.Constants.java` have to be added, according to the new sensor platform.

The SensorPlatform for the new sensor class can be started from the `startIntentServices()` method in `sensors.SensorManager.java` via an additional `Intent`, according to the existing platforms.

This also requires adding the Service for the new sensor platform as an additional `<service>` element in the app's `AndroidManifest.xml`.

SensorDataBus

The `SensorDataBus` class is a central component that acts as a broker for measurement data between the `SensorPlatform` implementations and components that register themselves with a `SensorDataBusListener` on the `SensorDataBus`.

Each of the registered listeners is informed about the new data in the `onDataAvailable()` method of the `SensorDataBusListener` interface. The notification of the listeners are executed in an own thread for each registered listener. This way, long lasting tasks in one listener will not block the notification of the other registered listeners. Within a listener, notifications are queued, until the handling of the previous notification is finished.

Data Storage

Local (SQLite)

The measurement data are stored locally in an SQLite database which is created on each startup with the current timestamp as filename. In other words, a new SQLite database is created every time a new measurement campaign is started.

After an ongoing logging process is stopped by clicking the `Stop` button in the UI, the remaining, cached items are saved to the SQLite database.

After stopping the logging, data are still provided from the SensorPlatforms to the bus, but it is no longer stored to disk. To start a new logging process, it is currently required to end the app's process and to restart it. This inconvenience will be addressed in a future version.

The SQLite storing is realised in `SQLiteStorage`, assisted by `SensorDataDbHelper`.

For the purpose of logging, `SQLiteStorage` registers a `SensorDataBusListener` on the `SensorDataBus` which puts new `SensorDataCollections` in a `BlockingQueue` that acts as a buffer between the listeners thread and the storage thread (created internally by `SQLiteStorage`). This storage threads puts the new data collections from the buffer queue to an `ArrayList` which is used to cache the measurements. Once this list has reached a defined size, the contained data are written to the current SQLite database in a transaction.

Additionally to the sensor data table in the SQLite database, tables for the platforms and sensors are created. These tables are dynamically populated, based the Enums which are used internally in the app and should therefore always be consistent.

Running Application in Emulator

Running the app in the emulator of Android Studio is possible, but it does make much sense, because Bluetooth devices are not supported. In order to run the app in the emulator the `build.gradle` file has to be extended by this snippet (see source):

```
android {
   splits {
          abi {
              enable true
              reset()
              include 'x86', 'armeabi-v7a'
              universalApk true
          }
     }
}
```

Documentation

The documentation of the eDiary app is created with Sphinx and uses the Read the Docs theme.

Installation:

```
pip3 install --user Sphinx sphinx_rtd_theme
```

The documents are written in ReStructuredText and can be converted to HTML and PDF using these commands:

```
make html; make latexpdf;
```

The creation of PDF requires a Latex installation. The created files can be found the folder `_build`. The website of the documentation is on: zgis.github.io/ediary-android-app/ . It can be updated by pushing the generated HTML to the branch `gh-pages`. It is very important to also include a file called `.nojekyll`, because otherwise GitHub will not publish folders starting with an underscore. However, the Makefile already creates this `.nojekyll` file automatically.

This script is useful for publishing the documentation source code:

```
make html
make latexpdf

WEBSITE_TARGET=../../ediary-android-app_website

cp -r _build/html/* ${WEBSITE_TARGET}
touch ${WEBSITE_TARGET}.nojekyll
cp _build/latex/eDiaryAndroidApp.pdf ${WEBSITE_TARGET}
```

Loading Data from Smartphone to Computer

The the source code of the ediary is an folder called `utility` which contains some useful commands for connection a computer with one or many smartphones. The documentation can be found directly inside the file.

Remove application folder on device

```
adb shell rm -rf sdcard/ediary
```

Backup eDiary data directly on phone

```
timestamp=$(date +"%Y-%m-%dT%H%M")

adb shell \
  mv \
  sdcard/ediary/sqlite \
  sdcard/ediary/${timestamp}_sqlite

adb shell ls sdcard/ediary/
```

Naming of the Phones

All phones are numbered. Their device name should be like *zgis_phone_4*.

Request balance of phone

- Dial `*121#` and the call, afterwards a SMS will be sent with the current balance. See this Link .

Switch Off Pin for SIM-card

`Settings -> Biometrics and Security -> Other Security Settings -> Set up SIM card lock`

BioHarness

BioHarness Download Section

3 Usage in Field

Some notes and best practices about using the eDiary app in practice.

Preparation

- charge all batteries
- Make sure you transport all devices, cables and accessories in proper bags (meshbags) or boxes
- ideally transport everything in a car
- on the test side, make sure you have enough space for all preparations e.g. a big table under a roof or a car, maybe even with electricity - ideally a room in a building
- bring laptop(s) for storing the data and checking
- store all data on the laptop and leave copies on the devices if there is enough space left
- check the weather forecast and adapt accordingly
- all devices have numbers. make sure participants always use the same combination of devices. This makes the data handling easier later on.
- Bring pen and paper and write down observations e.g. sensors that do not work
- Powerbanks might be useful for recharging the sensors

Data Organisation

- create a data structure before the field test
- each folder can have a text-file where the title contains the numbers of the sensors
- make sure the laptop has enough memory - consider bringing a external hard disc - ideally encrypt it
- after transfering the data from the sensors to the laptop, make sure to make the sensors empty. This causes less confusion later on.

GoPro

- screw driver might be necessary for adjusting the mounts
- battery lasts only 2h, bring replacement batteries
- set the required video settings. This link helps finding a good setting e.g. `720, 25fps`
- transfering the videos via cable is very errorness, consider bringing a MiniSD-card reader
- consider naming the the GoPros or at least the Mini-SD-cards
- consider writing a script that pulls the videos from GoPro (saves time)
- consider switching off the red light that indicates a record - it distracts other pedestrians

BioHarness

- bring wet wipes (a.k.a. Feuchttücher) for cleaning the devices
- also bring disinfectant spray for cleaning
- consider bringing extra BioHarness belts
- bring extra shoulder straps - might be more comfortable for some persons
- consider washing the belts, drying could be accelerated with a hair dryer
- have an own table for cleaning and drying - organize this table so that you always know which belts are ready, which ones are drying and which ones are dirty

Empatica E4

- make sure the device is tight enough in order to make good measurements
- It can cause troubles to connect different sensors at the same time and the same location. Therefore it is recommended to connect the sensors one after the other and also stay away from other Bluetooth devices (e.g. other smartphones).

Phone

- Battery typically lasts long enough
- mobile internet must be switched on in order to connect to E4, hence charge enough credit on the phone
- eventually bring phone credit sheets
- the latest stable version of the eDiary app should be installed
- delete previous measurements before the experiment
- adjust the config file
- make sure all settings on the phones are identical
- activate bluetooth, GPS
- activate wake lock (prevents falling asleep)

Storing the data later on

- create one directory with the raw data
- create another directory were the data is sorted
- this is redundant but makes sure no data is lost
- describe the data with proper README files

- make sure the data is properly backed up in the cloud (MyFiles, Uni Salzburg) or on an internal shared drive

Miscellaneous

- bring signs that people can find you
- bring enough pens for the written surveys
- bring clipboards and paper for notes
- prepare food and drinks for both employess and test persons
- there should not be too many people around, otherwise it is getting chaotic
- bring a laptop stance - this is more convenienet if you work long time
- bring post-its for quickly writing to notes to the sensors

4 Changelog

v.0.4.0 - 2020-01-21

Added

- German translation
- Start CHANGELOG using Keep a Changelog
- Permission dialogs for enabling bluetooth and location
- Publish documentation on zgis.github.io/ediary-android-app/
- SQLite filename contains name of the phone
- Optional location status info on main screen
- Survey: FreeText can be empty
- BioHarness: display connection and battery status
- BioHarness: support pairing from inside the app

Fixed

- Survey and Marker crashing the app when location is null
- Documentation with Sphinx can again export PDF, this time using Latex
- Realtime visualization should not crash anymore

v0.3.0-alpha - 2019-04-10

- Upgraded Empatica SDK to 2.2
- New implementation of Location tracking - should now work with Android 8
- Location is stored in an own table
- Added some bash and python utilities. These help to pull the data from the phone to the computer. Moreover some small charts are created.

v0.2.0 - 2018-10-05

- Adds an optional marker button
- Rename app to “eDiary”
- Adds visualization of real time measurements to the menu. **Warning** This feature crashes sometimes and is therefore **experimental**
- The survey and the visualization can be hidden via an entry in the configuration file.
- Adds the possibility to have a free text as survey item.
- Converts the dropdown menu of the `MultipleChoice` question type to a `RadioButtonGroup`(similar to a checkbox).

v0.1.0

Adds a dynamic survey to application. Before the questions were hard-coded. Now the questions can be specified in the XML config file. The app will create the database table and the GUI for the survey.
