# Supplementary figures and images for: An eDiary App Approach for Collecting Physiological Sensor Data from Wearables together with Subjective Observations and Emotions
Source: Sensors (Basel). 2022 Aug 16;22(16):6120. doi: 10.3390/s22166120 (PMC9414387; doi:10.3390/s22166120)

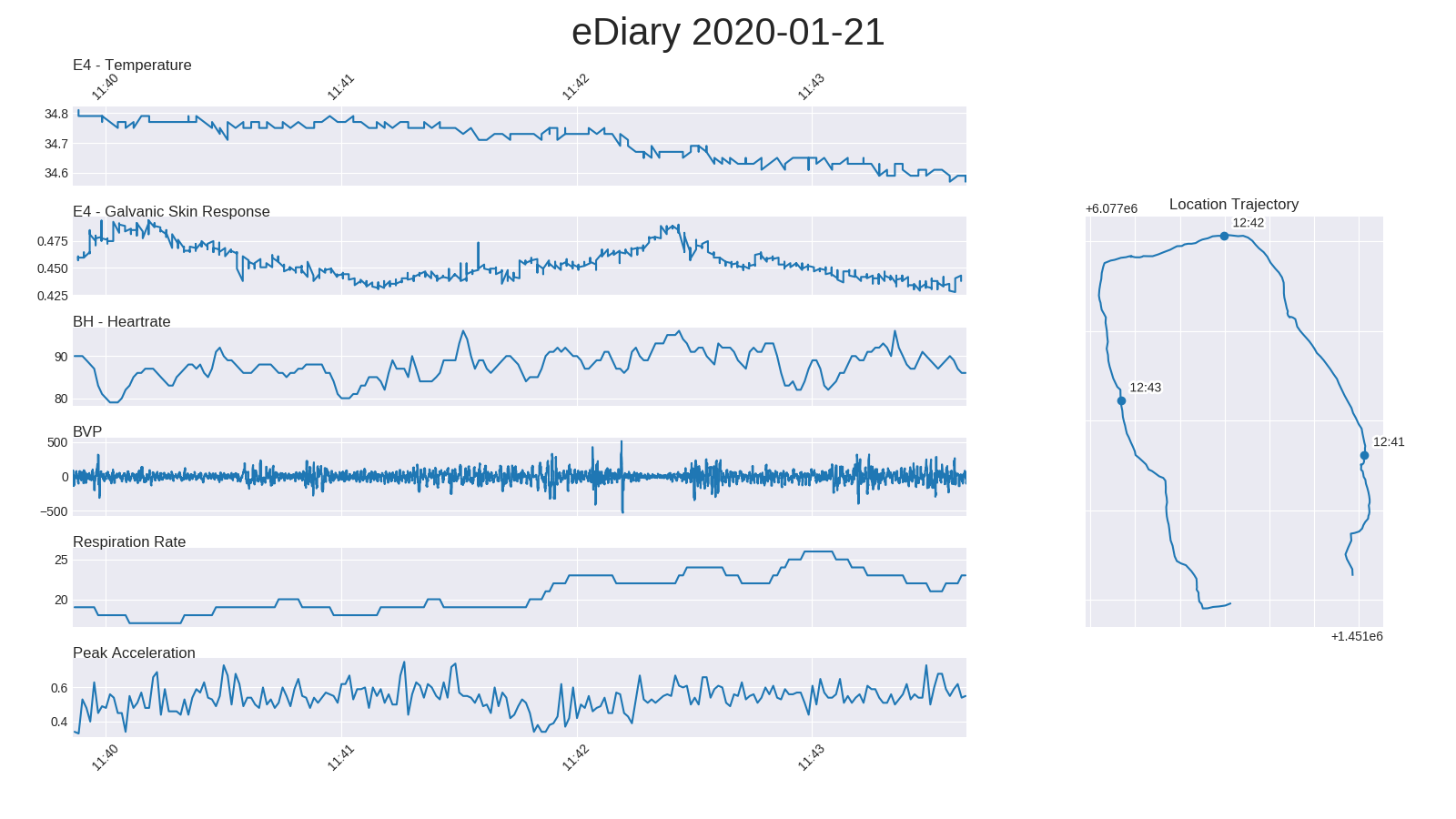

Supplement: Supplementary file 1 [file sensors-22-06120-s001.zip › supplementary_materials/v0.4.0_sample_data_chart.png]
